# An Adaptive Multilevel Security Framework for the Data Stored in Cloud Environment

**DOI:** 10.1155/2015/601017

**Published:** 2015-07-16

**Authors:** Sudha Devi Dorairaj, Thilagavathy Kaliannan

**Affiliations:** Coimbatore Institute of Technology, Coimbatore 641014, India

## Abstract

Cloud computing is renowned for delivering information technology services based on internet. Nowadays, organizations are interested in moving their massive data and computations into cloud to reap their significant benefits of on demand service, resource pooling, and rapid elasticity that helps to satisfy the dynamically changing infrastructure demand without the burden of owning, managing, and maintaining it. Since the data needs to be secured throughout its life cycle, security of the data in cloud is a major challenge to be concentrated on because the data is in third party's premises. Any uniform simple or high level security method for all the data either compromises the sensitive data or proves to be too costly with increased overhead. Any common multiple method for all data becomes vulnerable when the common security pattern is identified at the event of successful attack on any information and also encourages more attacks on all other data. This paper suggests an adaptive multilevel security framework based on cryptography techniques that provide adequate security for the classified data stored in cloud. The proposed security system acclimates well for cloud environment and is also customizable and more reliant to meet the required level of security of data with different sensitivity that changes with business needs and commercial conditions.

## 1. Introduction

**Figure 1:**
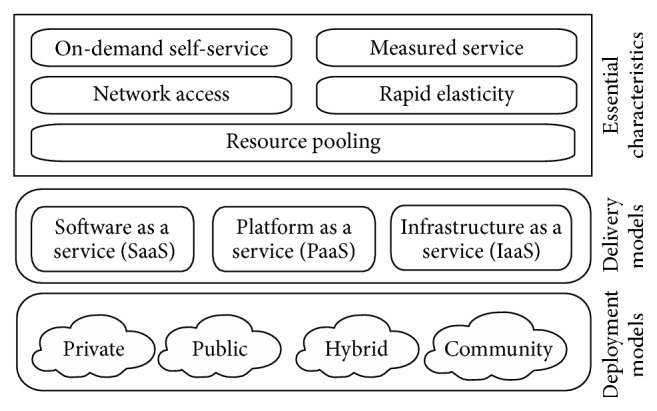
Model of NIST definition of cloud computing.

In today's global electronic village, cloud computing is emerging rapidly as a ubiquitous internet based computing service. The National Institute of Standard and Technology (NIST) defines it as “cloud computing is a model for enabling ubiquitous, convenient, on demand network access to a shared pool of configurable computing resources (e.g., networks, servers, storage, applications, and services) that can be rapidly provisioned and released with minimal management effort or service provider interaction” [1].  Figure 1 depicts the visual model of NIST definition of cloud computing.

The essential characteristics of cloud computing are on demand self-service, broad network access, measured service, rapid elasticity, and resource pooling. Cloud service model comprises Software as a Service (SaaS), Platform as a Service (PaaS), and Infrastructure as a Service (IaaS) and the deployment model includes private cloud, public cloud, hybrid cloud, and community cloud. Cloud computing requires strong security in its foundational elements like virtualization, distributed computing, SOA, broadband networks, application, data transmission, data storage, and so forth. These cloud fundamental elements require adequate security that varies with respect to the deployment model being used by the cloud user [2]. Among these elements, this paper attempts to give a probable solution for the security of data stored in public cloud.

Organizations usually focus on prime aspects of their business process and hence other subsidiary business processes that complicate the organization's functionality when handled internally are outsourced to an external service provider. Organizations opt for outsourcing to gain the most significant advantages like cost savings, focus on core activities, access to experience, improving performance, and flexibility [3]. In most of the organizations, business outsourcing is more common and they plan to leverage cloud computing since it facilitates organization to acquire both compute and storage resources on demand at reduced operational cost from the provider. Also, organizations need not to spend much on capital expenditure to own the necessary infrastructure internally to meet the fluctuating uncertain compute demand.

Despite the significant benefits, one sole aspect that hinders the adoption of cloud by many organizations is the data security challenge. Cloud Security Alliance's survey report states that “security remains the top barrier to cloud adoption.” Along with security challenge, the loss of control over IT services, concern over compromised accounts, insider threats, business continuity, and disaster recovery are the other top challenges holding back cloud projects [4]. Since the ever increasing voluminous and sensitive data of organizations are stored on shared servers at some third party's premises, the data owner lacks full control over the data and computation. Also, the data migrated to cloud is being accessed and shared by several internal and external users and hence it is highly important for an organization to defend their data from unauthorized access, use, and disclosure that may be detrimental to the organization. These data security threats force the data owners to provide adequate security in order to protect their sensitive data. Many of these security challenges must be addressed through a strong defending security management initiative that delineates the responsibility of both the service provider and the data owner.

As any single security method or common multiple security method for all data would be too costly or vulnerable for repeated attacks, this research suggests an adaptive multilevel security framework based on data sensitivity that manages to provide adequate level of security for the data classified under different classes. The proposed security system encompasses suitable access control combined with encryption techniques and a review control mechanism based on log analysis which facilitates reclassification of the data based on subsequent changes in the sensitivity levels of the data and changes in the security measures to meet the dynamic changes in cloud security threats.

The rest of the paper is organized as follows: Section 2 elaborates security challenges and measures to protect the data stored in cloud, Section 3 deals with proposed security framework, Section 4 discusses implementation and results, and Section 5 concludes with conclusion.

## 2. Security Measures to Protect Stored Data

### 2.1. Security Challenges to Stored Data in Cloud Environment

Cloud offerings are prominent and felicitous for today's business. In spite of the significant benefits, security is one main concern that hinders the adoption of cloud by many businesses and individuals. The top ten obstacles and opportunities for the growth of cloud computing are enunciated in [5]. Data confidentiality and auditability is one among the top ten obstacles. The problems in data confidentiality and data auditability are discussed in detail and probable solutions are suggested in [6]. Eltayeb has conducted a survey to understand user's acceptance on Personal Cloud Computing (PCC) and asserted that “in spite of the potential benefits of PCC, security and privacy risks are deterring many users from moving towards PCC” [7]. Cloud Security Alliance's Cloud Adoption Practices & Priorities Survey report states that “73 percent said concerns about the security of data are a top challenge holding back cloud adoption.” Also, it reveals that “while the security of data remains a top barrier to cloud adoption, organizations are still moving forward in adopting cloud services.” Since cloud has clear benefits, organizations opt for cloud services and in order to make use of it perfectly, organizations are in need to enforce the same security policies that they do for data stored on premises. In addition to the security challenges, one more concern to be noticed is the loss of control over IT services [4]. Since the data owner lacks full control over the outsourced data in cloud environment and the management of data in cloud storage may not be trustworthy, the migrated data may get exposed to various insider and outsider attacks. All cloud service providers usually engross their customers and hence protect their customer's data from malicious outsider attacks to the maximum extent. Therefore, despite concerns about the security of data, organizations are still moving forward in adopting cloud services. But the outsourced data may either accidently or intentionally get leaked by the insiders, that is, by the privileged users in the service provider's site. Malicious insiders' attacks that exist in the cloud system pose a serious threat to organizations. Insider attacks can be executed by malicious employees at the provider's site [8]. Sensitive data hosted in cloud on unauthorized disclosure or destruction can lead to significant business losses. Hence, it is mandatory for data owners, who move their data to cloud, to secure the valuable and critical data from their perspective.

### 2.2. Securing Outsourced Data at Rest

Organizations' data transactions are voluminous in nature but most of the time these data are at rest in the storage media. Lack of security makes these storage environments unreliable and unavailable which leads to considerable loss for the organization. Protecting data at rest is a critical aspect of information security. There are many security measures that can be taken including access control, logical separation, physical security, and encryption [9]. The importance of data security when using cloud environment at all levels is described in [10]. The best way to secure data at rest from malicious users is to fix and restrict the access to data. This can be achieved through access control which comprises authentication and authorization methods. Despite proper security provided through access control, still data breaches happen nowadays. So, a next level of security measure has to be provided to secure data at rest. Protecting data at rest is best handled through encryption methods. Encrypted data are self-defensive and enhance data security. A good security framework must encompass the four primary security measures, that is, access control, encryption, integrity verification, and log analysis. Along with these primary security measures, data classification is included as the fifth measure and is discussed in the following section.

### 2.3. Measure 1: Access Control

Access control ensures fine-grained access to resources and hence any security system cannot be designed without access control. Access control is a mechanism that controls the flow of data between subject (users, computers, applications, etc.,) and object (computers, applications, files, servers, devices, etc.,) where subject is an active entity that requests access to an object and object is a passive entity that contains the data. To prevent unauthorized access to data, either a single access control method or a combination of multiple methods is required. The importance of access control and its relationship to other security services are dealt in [11].

Among the different types of access control, Mandatory Access Control (MAC) is considered to be the strictest approach and is based on security level. In this model, the data are classified into different categories and each data has security labels based on classification and users also have classification property. Access is granted to users having the same classification property as that of the requested resources. To improve the access control model, Mandatory Access Control should be combined with Role Based Access Control (RBAC) which provides the concept of roles or separation of duties. Permissions are assigned to roles and then each user is assigned to a particular role. When a user changes his role, it is enough to revoke his role and no other changes are required. RBAC is a widely used access control model and also suits well for cloud environment [12, 13].

### 2.4. Measure 2: Data Encryption

Cloud Security Alliance [14] relates that, “for a security professional, it is obvious that if an organization needs to store data and does not trust those who can access or use the data, then the data must be encrypted.” Encryption is a perfect method for ensuring data confidentiality. Data classified as sensitive can be well protected using encryption techniques. Encryption is the process of encoding data in such a way that only authorized users can decode and use the data. Cryptography algorithms must be applied to data selectively according to the security requirement of that data which could make sense in using it. Data owners can select the right encryption schemes bearing in mind the required level of security and performance for the classified data. Following is the list of industry standard cryptography algorithms which can be applied for the classified data [15]:

- Advanced Encryption Standard (AES), 128, 192, or 256 bits,
- Rivest Cipher 6 (RC6), 256 bits,
- Blowfish, 128 or 448 bits,
- Triple Data Encryption Standard (Triple DES), 112 or 168 bits,
- Rivest Cipher 4 (RC4), 128,
- International Data Encryption Algorithm (IDEA), 128,
- Carlisle Adams and Stafford Tavares (CAST), 128,
- Rivest Cipher (RC5), 128,
- Secure and Fast Encryption Routine (SAFER), 128 bits,
- Rivest-Shamir-Adleman (RSA), minimum 1024 bits,
- Elliptic Curve Cryptography (ECC), minimum 384 bits,
- Symmetric Substitution and Transposition Techniques.

### 2.5. Measure 3: Integrity Verification

Data integrity helps in assuring the accuracy and consistency of data. Integrity is intended to ensure correctness of data upon later retrieval and to ensure that the data is the same as it was originally recorded. The Digital Signature Algorithms suggested in [15] could be used for verifying integrity of the classified data:

- Rivest-Shamir-Adleman (RSA), (minimum 1024 bits) with Secure Hash Algorithm-1 (SHA-1),
- Digital Signature Algorithm (DSA) (minimum 1024 bits) with Secure Hash Algorithm-1 (SHA-1),
- Elliptic Curve Digital Signature Algorithm (ECDSA) (minimum 384 bits) with Secure Hash Algorithm-1 (SHA-1).

As the algorithms have different processing times for different file sizes, data owners can select the algorithm according to their requirement.

### 2.6. Measure 4: Log Analysis

Log monitoring system is one of the important aspects of security. Logs are composed of log entries; each entry contains data related to a specific event that has occurred within a system or network [16]. Organizations create and maintain large volumes of log data for the purpose of continuous monitoring of security. This log data is used in analyzing security attacks either that happened or may happen in future. Regarding cloud, since the organization's sensitive data is hosted at some third party cloud servers, it is mandatory to include a log monitoring system in the security framework. Log entries must be routinely monitored for organizational activities and user behavior analysis. For the classified data stored in cloud, a review control mechanism must be used to find when and which data should be reassessed for its sensitivity value and need to reevaluate the adaptive security based on reassessed value. Also from the log analysis, obsolete data can be identified and isolated and can be stored in low cost storage servers. Another advantage of review control mechanism is, from the log analysis, the most frequently accessed data and the data that are rarely used can also be identified and appropriate encryption algorithm can be fixed to improve the performance.

### 2.7. Measure 5: Data Classification

The objective of data classification is to determine the required level of security for data and to protect data by providing adequate level of security according to the risk levels of data. Classification of data helps in determining the baseline security controls for safeguarding the data [17]. The organization's information system must be prudently examined and classified based on its level of sensitivity and the impact to the organization if the data are modified, disclosed, or destroyed without authorization [18]. Classification identifies and separates the most sensitive data from less sensitive data. In order to assess data and to determine the required level of security to protect each of the data, data classification standard is used.

The organization's data assessed with high or moderate or low levels of sensitivity are, respectively, categorized into high or moderate or low sensitivity classes. All data within a class are more or less with same level of sensitivity and hence can be protected with the same level of security. The protection given to data belonging to different classes with different levels of sensitivity by different security schemes ensures multilevel of security for the entire data of an organization. A combined approach that can efficiently protect data from the beginning to the end, that is, from the owner to the cloud and then to the user using classification, Secure Sockets Layer (SSL), Message Authentication Code (MAC), and searchable encryption is discussed in [19].

#### 2.7.1. Data Sensitivity

The first step to classify data is to determine its sensitivity based on the security objectives availability, integrity, and confidentiality (AIC) which could be followed by data classification. The importance of these security objectives for any data and its potential impacts are well defined in FIPS 199 Publication [20]. This model is referred to as AIC triad and is considered as the industry standard for computer security which is based on the three important characteristics of data which gives value for its use in organizations: availability, integrity, and confidentiality [21]. The data owner should monitor the data throughout its lifecycle and must discreetly analyze every data to identify the potential impact on unauthorized disclosure or destruction of that data. Based on the assessed levels of impacts due to loss of AIC, the sensitivity value of the data should be fixed.

#### 2.7.2. Classification Plan

The classification plan is to categorize the data into one of the three classes, namely, high, moderate, or low, based on the values assigned for the security objectives of that data. Following the data classification, appropriate required level of encryption methods should be selected and implemented to protect the classified data.

Table 1 depicts the AIC table with three classes: high, moderate, and low. The possible combinations of potential impact that any data may possess are shown. Since there are three parameters to be considered each with three impact values, 3^3^ combinations are possible as per the following hypothesis for AIC table formation:

1. Availability, integrity, and confidentiality of data can have minimum value as 1.
2. The value for the impacts high, moderate, and low can have values 3, 2, and 1, respectively.
3. Sensitivity value is determined by sum of values of all security objectives.

Using the hypothesis, the sensitivity value is determined which ranges from 3 to 9. However, the data can have different security objective value combinations, in general; otherwise, it could be concluded that the highest value among the security objectives should be fixed as the sensitivity level for that data. The following describes the algorithm to classify data using the hypothesis of the AIC table.

**Step 1 .**

Get the organization data.

**Step 2 .**

Analyze data to identify risks.

**Step 3 .**

Find the potential impact on organizational data which is either low||moderate||severe.

**Step 4 .**

Based on impact, fix 1||2||3 for A, I, and C for each data.

**Step 5 .**

Determine the sensitivity value.

**Step 6 .**

If sensitivity value = 7||8||9, then Class = I.

If sensitivity value = 4||5||6, then Class = II.

If sensitivity value = 3, then Class = III.

**Step 7 .**

Allocate the adequate security algorithm.

#### 2.7.3. Data Segmentation

**Figure 2:**
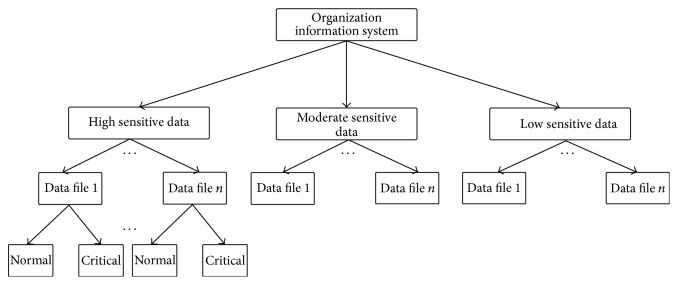
Classification and segmentation of organization information system.

Data are classified based on their sensitivity to the organization. The data in Class I are concluded to be highly sensitive which requires additional security. Hence, the data in Class I, based on its criticality, can be further segmented to form different partitions. Besides classification, segmentation of data makes the sensitivity get subsided further. To adhere this, the data owner has to identify significant break points and must segment the data into normal and critical segments. For the data segmented into normal and critical segments, suitable different security algorithms should be applied. The data classification of an organization information system with normal and critical data segments is shown in Figure 2.

## 3. Data Security Framework for Cloud Environment

### 3.1. Need for Data Owners to Protect Their Data

Cloud service providers deliver highly scalable cloud computing platform for users to build a wide range of applications. The pioneer of cloud computing, Amazon Web Services (AWS), recommends its customers to protect their data using appropriate methods before migrating into cloud environment. AWS Security Processes [22] state that “encryption of sensitive data is generally a good security practice, and AWS encourages users to encrypt their sensitive data via an algorithm consistent with your applicable security policy.” Even though the cloud service providers protect their customers' sensitive data hosted in their servers, any malicious insider may intentionally or accidently leak the sensitive data which leads to loss for the data owner. Thus, organizations or individuals hosting their valuable data in cloud are in an imperative need to have their own security schemes to protect their data that is being outsourced. For these reasons, an adaptive multilevel security management framework for the stored data is suggested in this paper that is more flexible and reliable which allows data owners to choose the appropriate combination of encryption schemes for different data in different classes along with standard authentication and access control mechanisms.

In the event of nonclassification of data, the data will be given the same security importance irrespective of the difference in sensitivity. Any uniform single encryption method for the entire information system proves to be costly if high encryption methods are used and security is compromised if simple encryption methods are used. Any common multiple security method encourages more attacks when the common security pattern is identified at the event of successful attack on any information. This necessitates identifying an adaptive multilevel security management framework.

### 3.2. An Adaptive Multilevel Security Framework for the Stored Data in Cloud Environment

The proposed security framework is a combination of security measures. As the proposed security framework is intended to protect the stored data in cloud environment, it encompasses four primary cryptography security measures, that is, access control, encryption, integrity verification, and log analysis. These renowned security measures must be customized while using in order to provide adequate security to the data hosted in cloud. Based on the nature of organization and its applications, any one or combination of security measures can be concentrated exhaustively. William Claycomb and Alex Nicoll have emphasized that “existing data protection techniques can be effective, if diligently and carefully applied” [23].

Protecting sensitive data is paramount for data owners. In the event, if such data are made public, the data owner has to face the consequences. Having data with different sensitivity levels, it is good to classify the data based on its sensitivity level. The term multilevel in this paper refers to the different security classifications based on the sensitivity of the data. Multilevel security or multiple levels of security is to classify data and protect the classified data by providing security at different levels starting with access permission based on security clearance which is followed by data encryption, integrity verification, and log analysis. Among the four primary security measures stated above, in this paper, classification followed by encryption is discussed exhaustively. Ensuring confidentiality and integrity of the data stored in an untrusted cloud environment can well be achieved by storing the data in encrypted form. Encryption solutions must be architected to achieve the goals of both data protection and availability as stated in [14].

A data owner may be an organization or an educational institution or any business or an individual who determines to host his data into cloud environment which will be utilized by the end users. Entities that consume the services provided by the data owners are referred to as end users. The reading/writing permissions granted to users depend upon the nature of applications that the organization possesses. For instance, in a digital library application, an owner has reading-writing access whereas the end users, that is, the subscribed readers, may have reading-only permission. Another example is a corporate with hierarchy which involves with several departments and projects that can be classified based on the security clearance. In this case, both the owner and the end user may be provided with reading-writing permissions where the end users are the employees of the organization.

**Figure 3:**
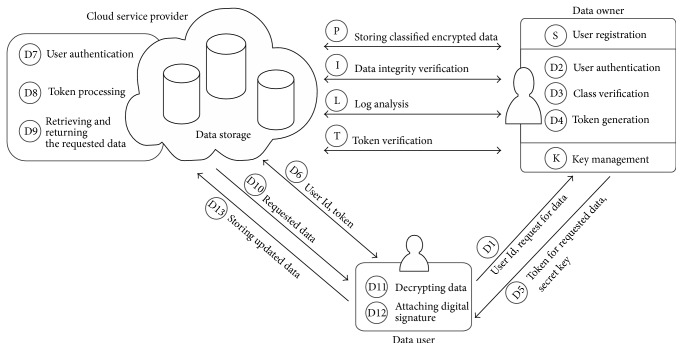
Overview of cloud security solution.

Figure 3 depicts the overview of the proposed security framework. The proposed model is focused on securing data at rest, that is, data stored in cloud storages. In this system, the cloud environment is used to store and retrieve the classified encrypted data by authorized users. So, the data owner outsources only the data and not the entire computations of an organization. The data owner has the opportunity to store the encrypted data in different sections of a bucket or in different buckets at the same location or at different locations as per the service level agreement made between the data owner and the cloud service provider.

The entities involved in this system are

  Data Owner (DO): an individual or an organization that outsources the classified encrypted data to the cloud environment. Data owner is responsible for user registration, user authentication, class verification for the requested data, token generation, key management, data integrity verification, and log analysis.
  Data Users (DU): authorized persons who request data and can use the data stored in cloud based on their access rights.
  Cloud Service Provider (CSP): it provides the storage service for data owners and allows the authorized users to retrieve the requested data after processing the access token submitted by the users.

*Preprocessing Phase (P)*. The following steps are carried out by DO:

1. Determining the sensitivity of data based on the security objectives A, I, and C.
2. Classifying the data based on sensitivity value into one of three classes: Class I || Class II || Class III.
3. Identifying the adequate security algorithm and encrypting the classified data with different encryption methods.
4. Storing the encrypted data in cloud storage.
5. Maintaining metadata for each data file which contains access privilege, classification type, and mapping data details.
6. Generating and maintaining secret key for the encrypted data.

*Phases of Cloud Security Solution*

*(I) Setup Phase (S)*

1. Data users (DU) register to the DO; DO categorizes data users based on the access rights assigned to them.

*(II) Data Accessing Phase (D)*

1. DU send request to the DO for accessing data.
2. DO verifies the user authentication and verifies whether the DU have access privilege to the requested data.
3. DO scrutinizes the request to identify to which class does the requested data belong to and appropriate levels of authentication verifications are done.
4. DO generates token from metadata for accessing the requested data.
5. DO sends the token and secret key to the DU.
6. DU submit only the credentials and token to CSP and retain secret key.
7. CSP verifies DU authentication.
8. CSP processes the submitted token to verify the access privilege.
9. CSP retrieves and returns the requested data.
10. DU download the requested data.
11. DU decrypts it using secret key.
12. Based on the access privilege, if DU perform any manipulations, then DU attach digital signature with the modified data.
13. DU store the modified data into cloud storage.

*(III) Key Management Phase (K)*. The proposed model assumes that the key stores of both DO and DU remain to be secured throughout the process. DO takes the responsibility of generation and distribution of secret keys to DU.

*(IV) Data Integrity Verification Phase (I)*. DO has to periodically verify the integrity of the data stored in cloud to check the correctness of data.

*(V) Log Analysis Phase (L)*. Using log monitoring system, data that needs to be reassessed for its sensitivity value and reevaluation of security measures are identified and are upgraded accordingly.

*(VI) Token Verification Phase (T)*. This step is executed between CSP and DO when there arise some disputes in the submitted credential and token for data accessing by the DU.

The data classified into Classes I, II, and III with suitable encryption methods are shown in Table 2. The types of access privileges and corresponding authentication levels are also elucidated.

#### 3.2.1. High Security Scheme for High Sensitive Class I Data

The data with high sensitivity value are grouped into Class I. As the data in Class I are highly important and should not be compromised, they require high level security. The high level security scheme for Class I is a combination of industry standard encryption and the strictest form of access control. The access control includes three-factor authentication for all users with reading and writing privileges. The authentication can be a combination of factors based on knowledge, token, and biometric schemes (e.g., password, security token, and biometric identifier). Based on the nature and criticality of the application, the data owner can use biometric authentication; or else it can be replaced with another token based authentication. For users with writing privilege, in addition to the three-factor authentication, digital signature of the user is to be attached for authorization and integrity verification. Whenever a user requests data access for Class I data, a One-Time Password (OTP) authentication must be used as an additional security measure. The user should submit the received OTP for further processing. OTP helps to verify the legitimacy of the user and also in general improves the security to one more level [24].

#### 3.2.2. Medium Level Security Scheme for Moderate Sensitive Class II Data

Data with a combination of moderate and low security objective values are fixed with the highest sensitivity value that is moderate when compared to low and hence such data are grouped into Class II. All data of Class II are with moderate sensitivity and hence to be protected with medium level security scheme. Medium level security is a combination of industry standard encryption and a stricter form of access control. The industry standard encryption methods that are not used for Class I may be used for Class II data because both Class I and Class II data if compromised lead to significant loss to the business. The access control includes two-factor authentication for all users with reading and writing privileges. The authentication can be a combination of factors based on knowledge and token (e.g., password, security token). For users with writing privilege, in addition to the two-factor authentication, digital signature of the user is to be attached for authorization and integrity verification.

#### 3.2.3. Base Level Security Scheme for Low Sensitive Class III Data

All data with low sensitivity level are grouped under Class III. All data of Class III require only base line security. Base line security is a combination of base level access control security and simple encryption security for the entire data of this class. Single factor authentication (e.g., password or personal identification number) is suggested to access data in Class III. For users with writing privilege, in addition to the single factor authentication, digital signature of the user is to be attached for authorization and integrity verification. Since all data of Class III are with low sensitivity, it involves only small risk when compromised and a simple encryption for the entire data is sufficient.

### 3.3. Security Analysis

From the security perspective, this approach offers several benefits. First, the confidentiality of the data is achieved since all the computations are managed by the data owner and the cloud contains only the encrypted data. Second, the levels of authentication verification and token generation are based on the type of requested data which constricts the access privilege and thereby holds the fine-grained access control. Third, user revocation process is easy by deleting the awarded user credentials without affecting other processes and if necessary secret key value can be changed by the data owner. Fourth, since different encryption schemes are used for different classes, the security is enhanced and the malicious users cannot predict the security pattern used for the stored data in cloud, whereas this is not possible in the event of using any single security method or any common multiple security methods. Finally, the suggested security system cannot be compromised by malicious insiders since the cloud storage contains only the encrypted data and also no adversary can compromise keys since they are not stored in cloud storage.

## 4. Implementation and Results

### 4.1. OpenStack Private Cloud

Public cloud offerings are very prominent solutions for the IT industry today. As public cloud is proprietary, users like researchers and students could not customize it to the maximum extent according to their requirements. Hence, private cloud is a treat for academic researchers and students to savvy cloud environment. A private cloud environment can be implemented in an institution using the widely available open source software to experience the cloud environment and to carry out the experiments. OpenStack is one such open source cloud operating system software which is customizable and hence students and researchers could experience the cloud environment, explore the algorithms used in cloud, and can use it as a real test bed for implementing their work.

**Figure 4:**
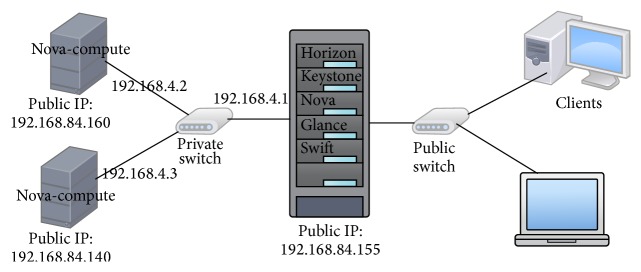
OpenStack private cloud setup.

A private cloud environment is deployed in our institution with three systems, Server1, Server2, and Server3, and a client system to interact with the deployed cloud servers. Server1 runs all the components of Nova, Glance, Swift, Keystone, and Horizon. Server2 and Server3 run only the Nova-compute component. One or more client systems can interact with the deployed OpenStack components as shown in Figure 4 [24].

The private cloud is deployed using OpenStack cloud operating system for testing and research purposes. A study on virtualization technique [25] is mandatory which helps to understand the basis of cloud environment. Initially, the private cloud setup functioning is tested by creating several VMs and software was installed. The software installed in the guest OS is readily accessible from anywhere within the institution campus.

In this paper, OpenStack cloud environment is used to test one of the four primary security measures, that is, encryption. Security measures discussed in Sections 2.4 and 2.7 are implemented here. One significant method to increase data protection, confidentiality, and integrity is to ensure that the data is protected within the cloud using encryption [26]. In the overview of cloud security solution (Figure 3), the data owner stores the classified encrypted data in cloud storage. For encrypting data files of different classes, different encryption algorithms to be used are suggested in Table 2. Since the data to be hosted is assumed to be voluminous, an analysis is imperative to find which industry standard algorithm works fine in the cloud environment. Hence, data files ranging from 16 MB to 260 MB in size are encrypted and hosted in the instance of OpenStack and are accessed from a remote client system. These data files are encrypted with different symmetric encryption algorithm. The results of the performance analysis between various symmetric encryption algorithms are discussed in detail in Section 4.2.

**Figure 5:**
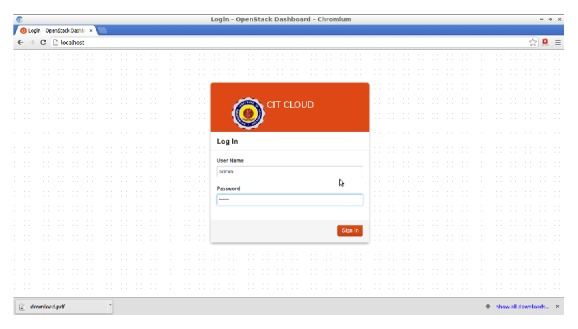
OpenStack login screen.

**Figure 6:**
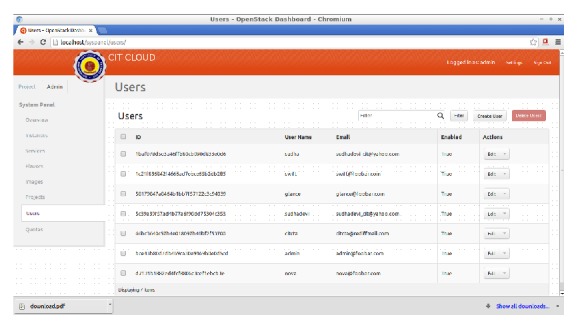
OpenStack user screen.

**Figure 7:**
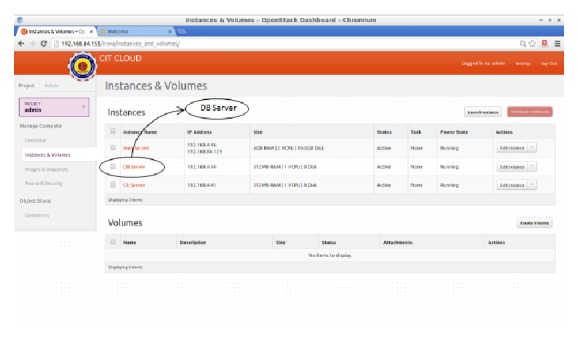
Instances created in OpenStack.

**Figure 8:**
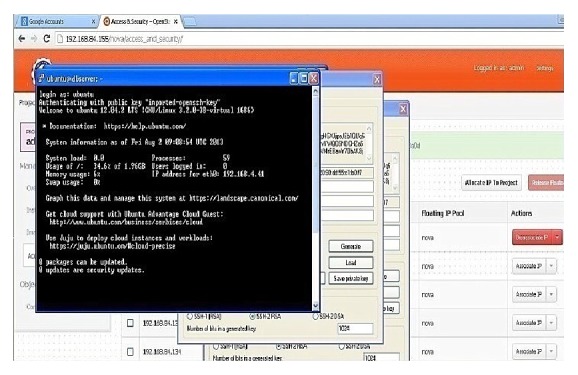
Accessing virtual machine from client node.

**Figure 9:**
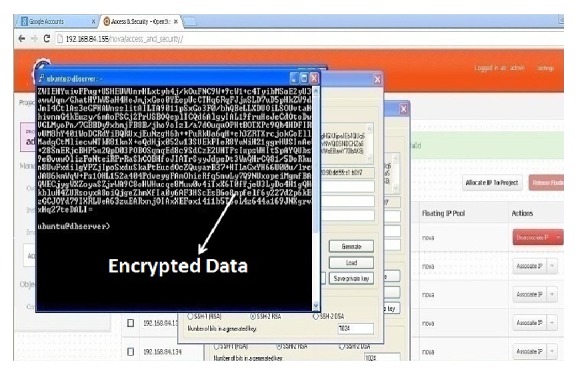
Encrypted data stored in cloud instance.

Figures 5–9 reveals the screenshots of the implementation.

Figure 5 depicts the login screen where cloud user enters the username and password for validation.

Figure 6 shows the various users having credentials to use OpenStack cloud.

In Figure 7, various instances created are shown. For each instance, an IP address is allocated. Three instances, namely, web server, DB server, and cit server, are created to serve different purpose for the institution. In the DB server instance, data files ranging from 16 MB to 260 MB in size are hosted. These data files are encrypted with different industry standard symmetric encryption algorithm as listed in Section 2.4.

Figure 8 depicts the interaction of a client system with the OpenStack instance.

Figure 9 displays the encrypted data stored in OpenStack instance that is accessed from the client node. That is, the encrypted data is stored in cloud storage by the data owner and the data user accesses this encrypted data from the client system.

### 4.2. Performance Analysis of Cryptographic Algorithms

**Figure 10:**
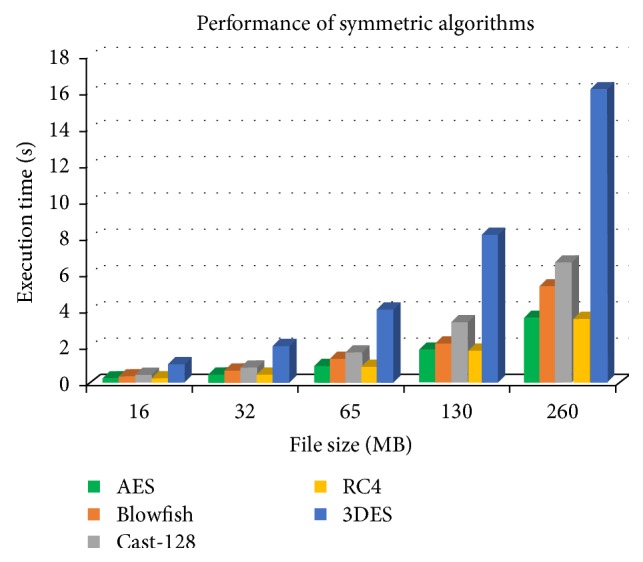
Performance analysis of symmetric algorithms.

The goal is to protect the data stored in cloud. Encryption is one of the most effective primary data protection controls used today [14]. Encryption algorithms differ from one another by means of several factors which includes the ability of encryption algorithms to protect information against malicious attacks and the time taken in doing so. Protecting data at rest can be best handled with symmetric algorithms because it is more suitable while considering performance demands. For most of the applications that deal with bulk data, symmetric algorithms are considered to be more efficient regarding performance and manageability than asymmetric algorithms [9]. References [27, 28] deal with many popular symmetric, asymmetric, and authentication algorithms. Among these algorithms, this section discusses the performance comparison between the most common industry standard symmetric encryption algorithms: AES, Blowfish, 3DES, CAST128, and RC4. The comparison factor considered here to evaluate the performance of various algorithms is the speed of the algorithm to encrypt and decrypt bulk data files of various sizes.  Figure 10 shows the performance analysis of symmetric algorithms compared in this section.

The stream cipher algorithm RC4 and the block cipher algorithm AES show better performance compared to other encryption algorithms. But since block cipher algorithms are more suitable for bulk data than stream cipher, it is concluded that AES algorithm is perfect for data files that are relatively large in volume. From the graph (Figure 10), it is obvious that AES suits best for cloud applications that require high security with relatively large data size. Also the graph shows that as the size of the data file increases, the performance of AES algorithm seems to be better compared to other algorithms.

## 5. Conclusion

Cloud is well known for its prominent offerings. Organizations interested in data outsourcing opt for cloud storages which satisfy the dynamic business requirements on demand. In spite of significant benefits, concerns about data security hold back cloud adoption widely. This paper proposes a multilevel security framework that is adaptive for cloud environment. The adaptive multilevel security framework proposes to classify the data based on sensitivity and to provide the appropriate required level of security to the classified stored data which is a pioneering way to improve and enhance dependent security in cloud environment. The ultimate goal of this adaptive multilevel security framework is to overcome the drawbacks of any single security method or any common multiple security method for the entire data with different sensitivity which is not a prominent solution.

The proposed security framework is based on multilevel approach to secure the stored data in cloud. Firstly, the sensitivity of the data is assessed and the data are classified and segmented accordingly if needed. Secondly, the data is protected from malicious users by providing enciphering methods based on the criticality and sensitivity of the data. Thirdly, the sensitive data is protected from unauthorized access by the inclusion of Mandatory Access Control that includes multifactor authentication based on classification. Finally, all authorized and unauthorized access are recorded in the log register which can be analyzed for predicting the attacks, taking control measures based on the attempted attacks by reclassification, and upgrading the security measures to suit the changing sensitivity of the data according to the business needs. The overall conclusion is that the proposed security framework acclimates with the dynamic cloud environment and is a promising approach to protect the data stored in cloud servers.

## 6. Future Work

In this paper, data classifications followed by different encryption methods are illustrated exhaustively. Access control and key management must be carefully collaborated to preserve the benefits of encryption techniques, because if the secret keys are compromised, there is no meaning in possessing data in encrypted form. That is, if the keys are compromised, then the encrypted data is compromised. So, literature survey on key management should be thoroughly articulated and probable solutions must be defined. Since data classification may involve hierarchical structure, there is an imperative need to design a novel hierarchical based key management scheme for the data stored in cloud environment. An efficient way to design hierarchical access control is to use Elliptic Curve Cryptography (ECC) which has recently received significant attention due to its high performance, low computational cost, and small key size [29]. Hence, a novel hierarchical access control scheme based on ECC is to be designed to fulfill security requirements in cloud environment.

## Figures and Tables

**Table 1:** AIC table.

C	High			Moderate			Low		
I	H	M	L	H	M	L	H	M	L
A									
H	9	8	7	8	7	6	7	6	5
M	8	7	6	7	6	5	6	5	4
L	7	6	5	6	5	4	5	4	3

**Table 2:** Multilevel security system.

Classification	Class I		Class II	Class III

Sensitivity	High		Moderate	Low

Level of security	High		Moderate	Base

Access control				
Operation and authentication				
Read	Three-factor authentication (e.g., password, security token, and biometric identifier)		Two-factor authentication (e.g., password and security token)	Single factor authentication (e.g., password or personal identification number)
Write	Three-factor authentication + signature + OTP		Two-factor authentication + signature	Single factor authentication + signature

Encryption security				
Segment	Normal data	Critical data, mapping data	The entire data	The entire data
Algorithm	Simple encryption (e.g., symmetric substitution algorithm)	Industry standard encryption (e.g., AES, RC4 algorithm)	Industry standard encryption (e.g., blowfish algorithm)	Simple encryption (e.g., symmetric substitution algorithm)
